# Novel *FAM83H* mutations in patients with amelogenesis imperfecta

**DOI:** 10.1038/s41598-017-05208-0

**Published:** 2017-07-20

**Authors:** Wang Xin, Wang Wenjun, Qin Man, Zhao Yuming

**Affiliations:** 1 Department of Pediatric Dentistry, Peking University School and Hospital of Stomatology, Beijing, PR China; 2 National Engineering Laboratory for Digital and Material Technology of Stomatology, Beijing, PR China; 3 Beijing Key Laboratory of Digital Stomatology, Beijing, PR China

## Abstract

Amelogenesis imperfecta (AI), characterized by a deficiency in the quantity and/or quality of dental enamel, is genetically heterogeneous and phenotypically variable. The most severe type, hypocalcified AI, is mostly caused by truncating mutations in the *FAM83H* gene. This study aimed to identify genetic mutations in four Chinese families with hypocalcified AI. We performed mutation analysis by sequencing the candidate *FAM83H* gene. Three novel mutations (c.931dupC, p.V311Rfs*13; c.1130_1131delinsAA, p.S377X; and c.1147 G > T, p.E383X) and one previously reported mutation (c.973 C > T, p.R325X) in the last exon of *FAM83H* gene were identified. Furthermore, constructs expressing Green fluorescent protein (GFP)-tagged wild-type and three novel mutant FAM83Hs were transfected into rat dental epithelial cells (SF2 cells). Wild-type FAM83H-GFP was localized exclusively in the cytoplasm, especially in the area surrounding the nucleus, while the mutant FAM83H-GFPs (p.V311Rfs*13, p.S377X, and p.E383X) were localized predominantly in the nucleus, with lower levels in the cytoplasm.

## Introduction

Dental enamel is the most highly mineralized tissue in the human body. Unique among the mineralized tissues, it is produced by ameloblasts, which have an epithelial origin. Enamel formation is a remarkably complex biomineralization process that is controlled by the regulated expression of many genes^1^. Studies have indicated that mutations in many of the genes can lead to amelogenesis imperfecta^2, 3^.

Amelogenesis imperfecta (AI) represents a group of inherited disorders that are clinically heterogeneous and exhibit enamel defects with or without systemic manifestation^4, 5^. Hypocalcified AI is the most severe form, in which the malformed enamel is cheesy-soft and stained, often abrading soon after tooth eruption. Mutations in *FAM83H* gene have been shown to cause autosomal-dominant hypocalcified AI (ADHCAI)^6–8^. Originally, the genes encoding enamel extracellular matrix proteins and proteases were primary candidates for AI, such as *AMELX*, *ENAM*, *AMBN*, *AMTN*, *MMP20*, and *KLK4* ^9–13^. *FAM83H* is the first-known gene encoding an intracellular protein that is associated with AI^6^. To date, more than 20 genes have been proven to be candidate genes in AI^14–22^. However, defects in *FAM83H* account for more AI cases than any other single gene^23^.

*FAM83H* is not specifically expressed in teeth, but is expressed in many tissues, such as the larynx, cervix, and bladder. Despite the relatively high prevalence of AI cases caused by *FAM83H* mutations, its function in enamel formation remains obscure. Based on bioinformatic analysis and structure and domain predictions, the characteristics of FAM83H show little indication of its potential function. Recently, some studies demonstrated that FAM83H might act as a scaffold and could regulate keratin cytoskeleton organization^24, 25^.

In the present study, we recruited four families with ADHCAI and identified three novel mutations (p.V311Rfs*13, p.S377X, and p.E383X) and one previously reported (p.R325X) *FAM83H* mutation causing enamel hypocalcification. The intracellular localization of the three novel mutant FAM83Hs were altered.

## Results

### Clinical and genetic findings

**Figure 1:**
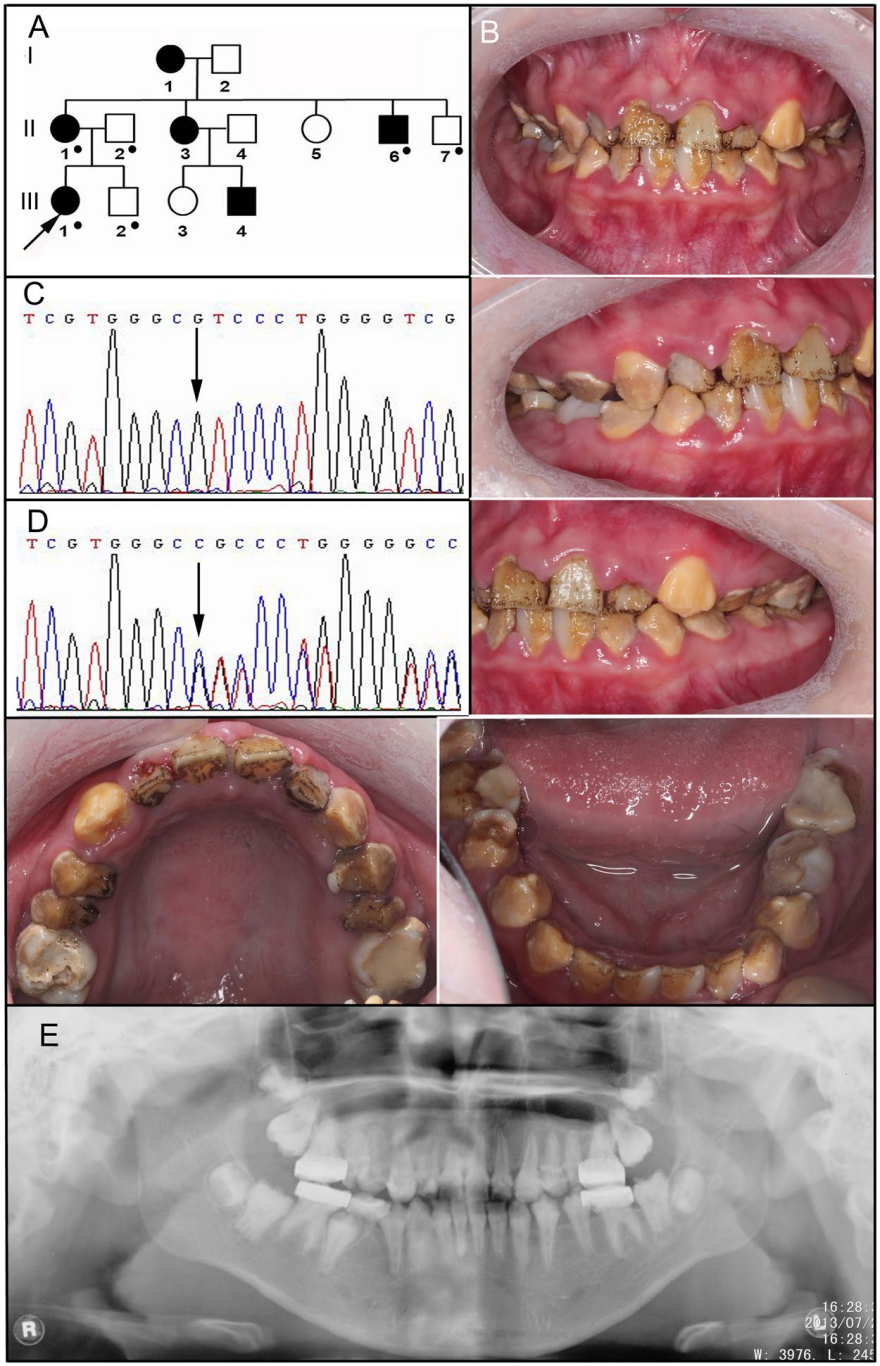
Clinical and mutational analysis of Family 1. (**A**) Pedigree of Family 1, Proband (III:1). Black dots indicate members recruited for this study. (**B**) Oral photographs showing enamel malformations categorized as hypocalcified AI. (**C**,**D**) *FAM83H* exon 5 sequencing chromatogram of an unaffected family member (II:7) (**C**), and the proband (III:1) (**D**), revealing a novel frameshift mutation: c.931dupC, p.V311Rfs*13. (**E**) Panoramic radiograph of the proband taken at the age of 11.

The proband of Family 1 was an 11-year-old girl who presented to our department with dental discolouration. Her enamel was generally rough and yellowish-brown, but normal-looking enamel could be identified in two lower incisors and two upper first permanent molars. According to her mother’s report, the enamel defects were inherited from the maternal grandmother (I:1). The proband’s mother (II:1), one aunt (II:3), one uncle (II:6), and one cousin (III:4) were all affected, indicating that the dental malformations were caused by a dominant mutation (Fig. 1A,B). Two affected family members (II:1, II:6) had restored their teeth with many dental crowns, but their unrestored teeth showed defective enamel (Fig. S1). Radiographically, the enamel layers of the proband were generally thin and less radio-opaque than the underlying dentin. Based on the enamel phenotype and the dominant pattern of inheritance, a diagnosis of hypocalcified AI was made, which implicated *FAM83H* as the possible pathogenic gene. By direct sequencing, we discovered a novel *FAM83H* frameshift mutation (c.931dupC, p.V311Rfs*13) that segregated with the disease phenotype (Fig. 1C–E).

**Figure 2:**
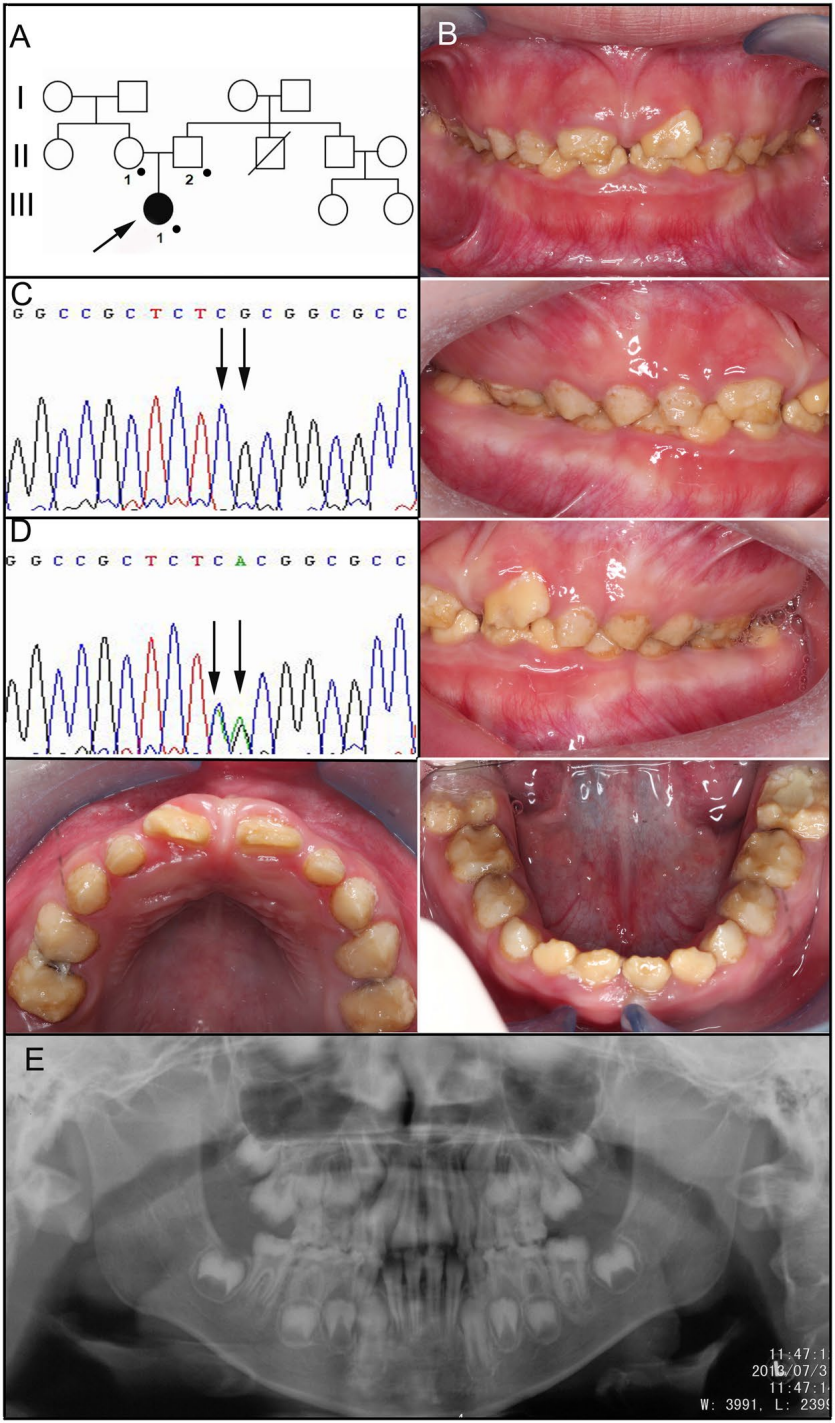
Clinical and mutational analysis of Family 2. (**A**) Pedigree of Family 2, Proband (III:1). Black dots indicate members recruited for this study. (**B**) Oral photographs showing enamel malformations categorized as hypocalcified AI. (**C**,**D**) *FAM83H* exon 5 sequencing chromatogram of an unaffected family member (II:1) (**C**), and the proband (III:1) (**D**), revealing a novel nonsense mutation: c.1130_1131delinsAA, p.S377X. (**E**) Panoramic radiograph of the proband taken at the age of 8.

The proband of family 2 was an 8-year-old girl who was the only affected individual in the family (Fig. 2A). The enamel of her mixed dentition was yellow-brown discoloured and cheesy-soft. The defective enamel chipped off two erupting upper incisors, which made them lack a normal contour. Despite the irregular rough tooth surfaces, proband 2 showed relatively sound gingival condition (Fig. 2B). The panoramic radiograph showed that the enamel of unerupted teeth appeared to be of normal thickness but did not contrast with the dentin. We screened the entire coding region and adjacent intron boundaries of the *FAM83H* gene directly, and identified a novel heterozygous nonsense mutation (c.1130_1131delinsAA, p.S377X) (Fig. 2C–E). The mutational analysis confirmed the clinical diagnosis and revealed that the enamel defects in the proband were caused by a spontaneous de novo mutation in *FAM83H*.

**Figure 3:**
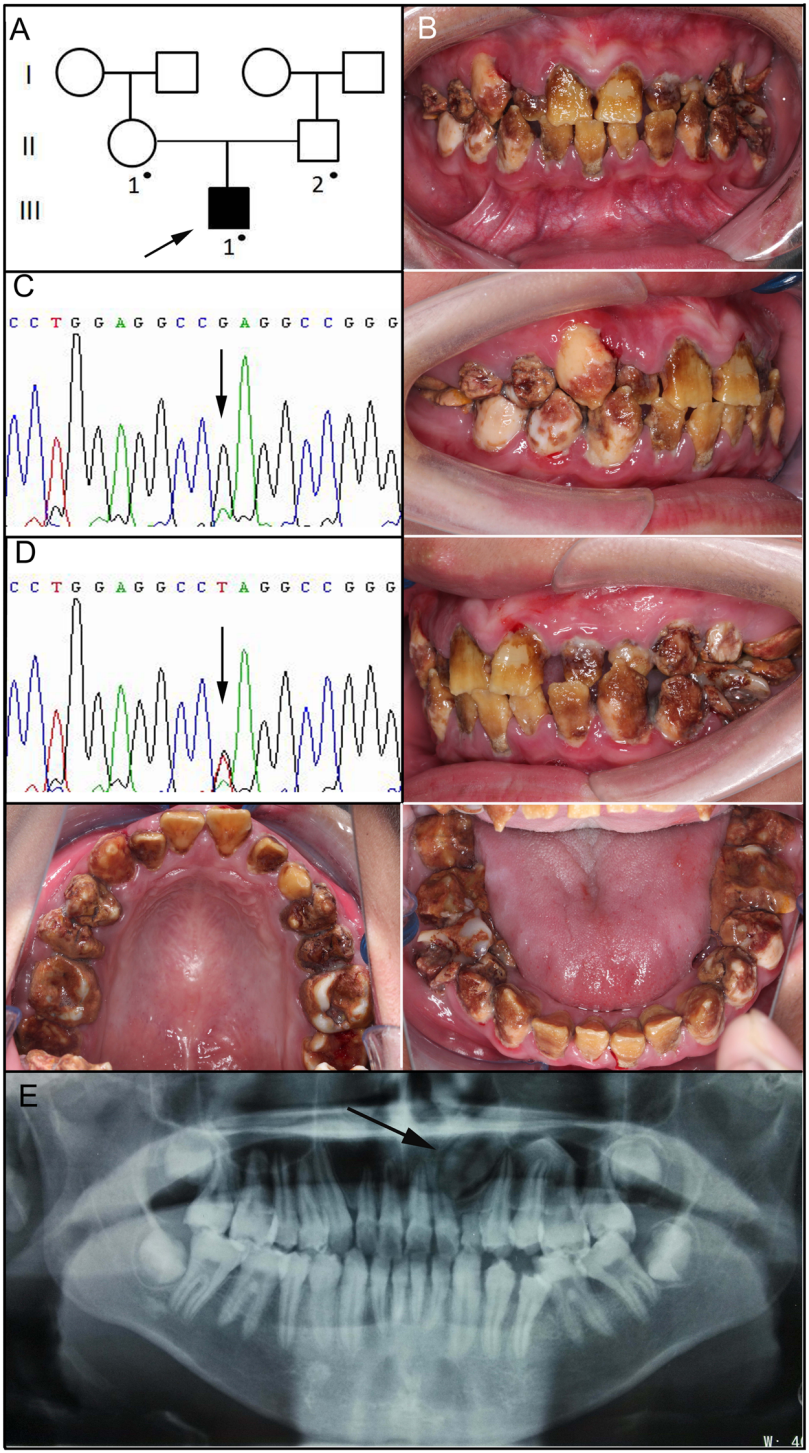
Clinical and mutational analysis of Family 3. (**A**) Pedigree of Family 3, Proband (III:1). Black dots indicate members recruited for this study. (**B**) Oral photographs showing enamel malformations categorized as hypocalcified AI. (**C**,**D**) *FAM83H* exon 5 sequencing chromatogram of an unaffected family member (II:1) (**C**), and the proband (III:1) (**D**), revealing a novel nonsense mutation: c.1147 G > T, p.E383X. (**E**) Panoramic radiograph of the proband taken at the age of 16. The arrow indicates the impacted upper left canine.

The proband of family 3, a 16-year-old boy, presented to our department with dental discolouration and sensitivity to thermal changes. He was also the only affected individual in the non-consanguineous family (Fig. 3A). His enamel was dark brown, and had chipped off from most of the incisors, whose incisal 1/2 was left particularly thin. Large amounts of dental calculus were accumulated on the irregular rough tooth surfaces, resulting in severe generalized gingivitis (Fig. 3B). The proband had an anterior cross bite and an impacted upper left canine, which was shown on the radiograph. Target gene mutational analysis revealed a novel *FAM83H* nonsense mutation (c.1147 G > T, p.E383X), which confirmed the diagnosis of hypocalcified AI (Fig. 3C–E).

**Figure 4:**
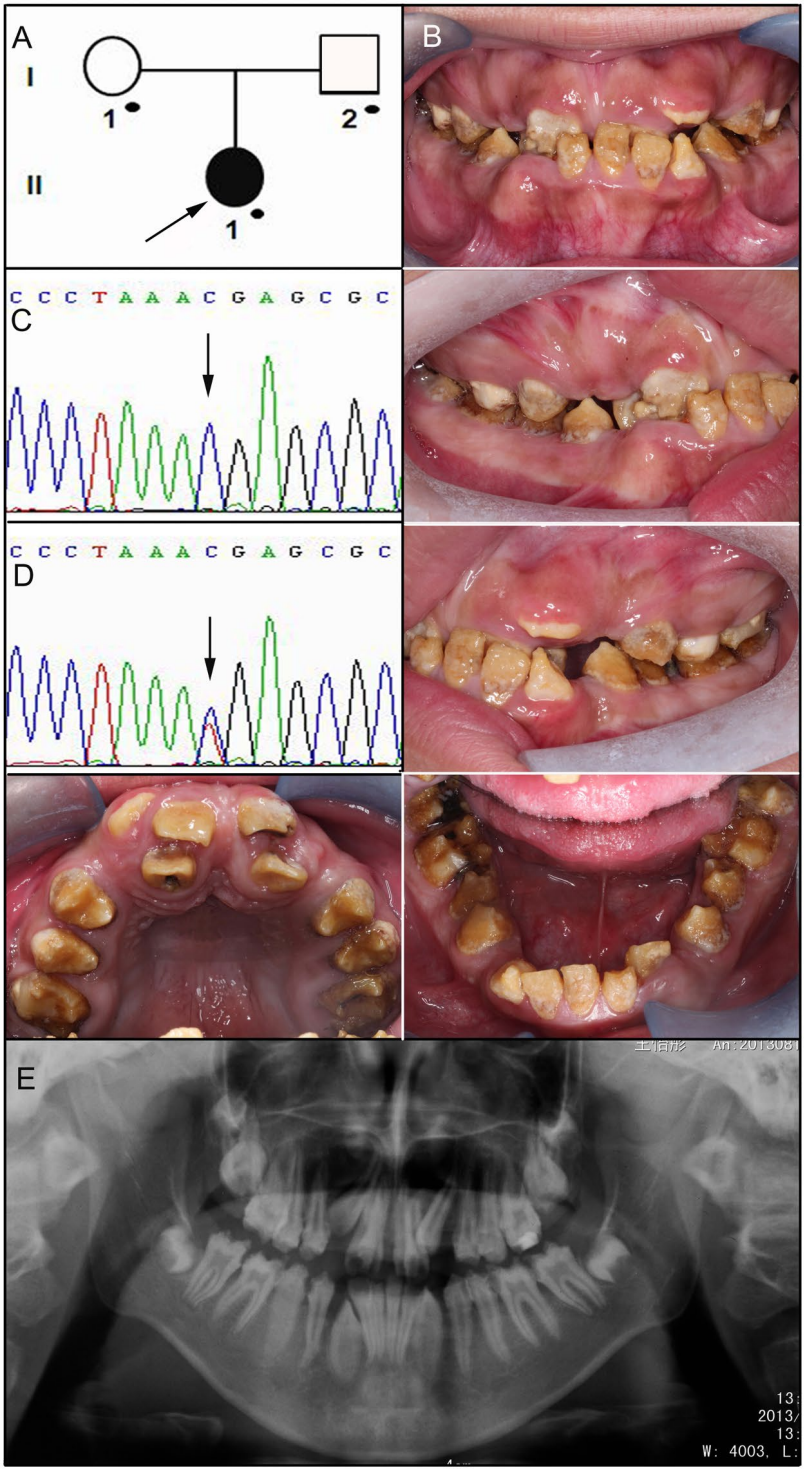
Clinical and mutational analysis of Family 4. (**A**) Pedigree of Family 4, Proband (II:1). Black dots indicate members recruited for this study. (**B**) Oral photographs showing enamel malformations categorized as hypocalcified AI. (**C**,**D**) *FAM83H* exon 5 sequencing chromatogram of an unaffected family member (I:1) (**C**), and the proband (II:1) (**D**), revealing a previously reported nonsense mutation: c.973 C > T, p.R325X. (**E**) Panoramic radiograph of the proband taken at the age of 14.

The enamel of proband 4 showed similar defects to those of the probands from the above three families. However, she exhibited a relatively complex malocclusion, including anterior cross bite, severe dental crowding, and ectopic eruption (Fig. 4A,B). The radiographic density of the enamel was lower than the underlying dentin. Therefore, mutational analysis was performed by amplifying all of the coding regions and splice junctions of the candidate *FAM83H* gene. One previously reported disease-causing mutation (c.973 C > T, p.R325X) was identified (Fig. 4C–E).

### Intracellular localization

**Figure 5:**
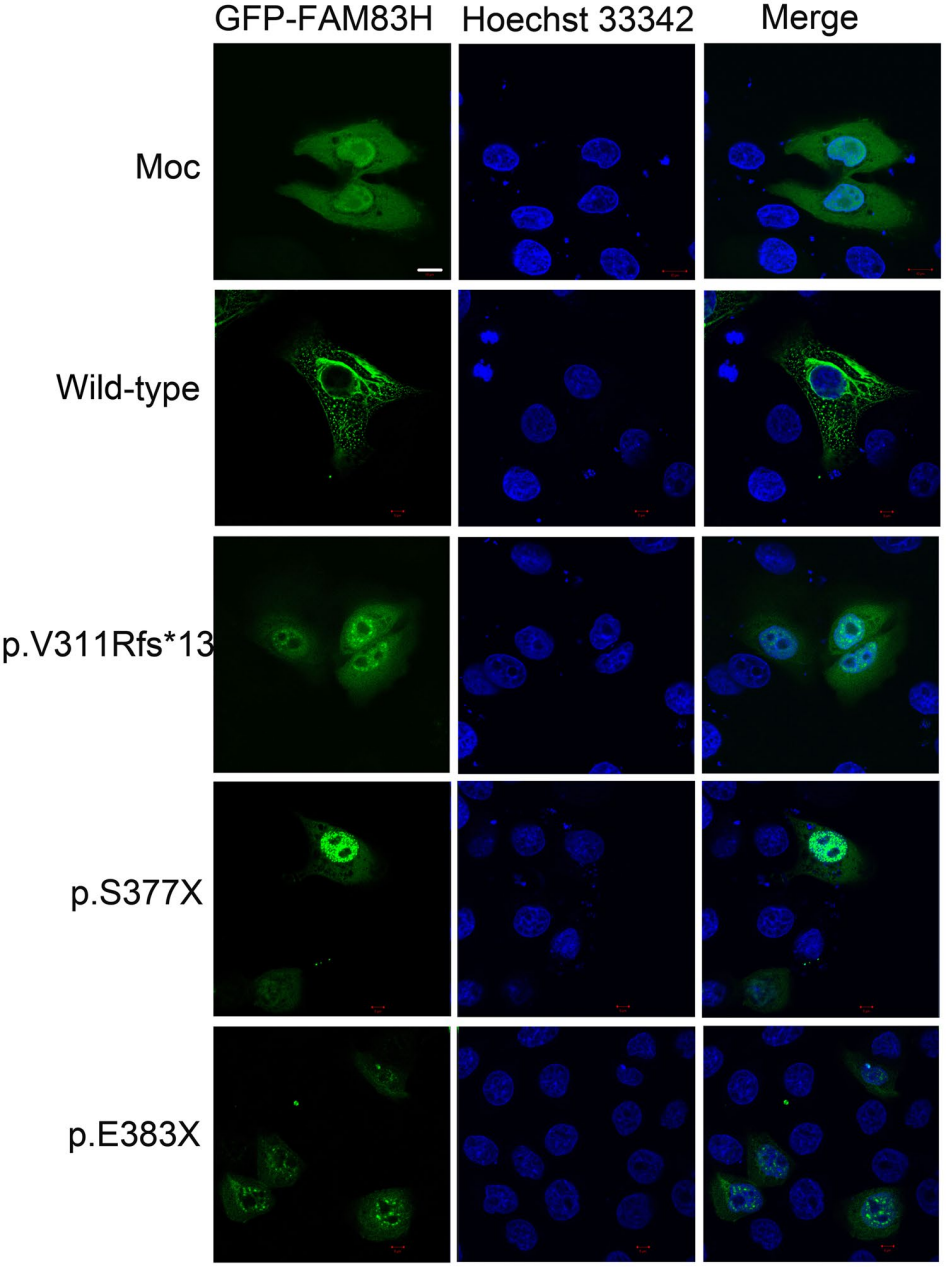
Subcellular localization of wild-type and mutant FAM83H-GFP in SF2 cells. Wild-type FAM83H-GFP was localized exclusively in the cytoplasm, especially the area surrounding the nucleus. The expression patterns around the nucleus and in the cytoplasm were present as filamentous and speckle-like shapes. The mutant FAM83H-GFPs (p.V311Rfs*13, p.S377X, p.E383X) were accumulated predominantly in the nucleus with lower levels in the cytoplasm, and their expression pattern was changed into speckle-like shapes. Nuclei were stained with Hoechst 33342.

The wild-type FAM83H-GFP fusion protein, expressed from vector pEGFP-C1, was localized exclusively in the cytoplasm, especially the area surrounding the nucleus. The expression patterns around the nucleus and in the cytoplasm were observed as filamentous and speckle-like shapes (Fig. 5). However, three mutant FAM83H-GFP proteins (p.V311Rfs*13, p.S377X, and p.E383X) were accumulated predominantly in the nucleus with lower levels in the cytoplasm, and their expression patterns were changed into speckle-like shapes (Fig. 5).

## Discussion

In the present study, we reported four *FAM83H* mutations, among which three (c.931dupC, p.V311Rfs*13; c.1130_1131delinsAA, p.S377X; c.1147 G > T, p.E383X) were newly identified. Consistent with previous studies, the novel mutations were all in the last exon of the *FAM83H* gene leading to a premature termination codon^26^. Among the genes that cause AI, *FAM83H* causes the highest percentage of cases and the most severe enamel defects. The genotype-phenotype correlation in predicting the causative gene in such cases has proved useful because of the association of hypocalcified enamel with mutations in *FAM83H*.

Clinically, dental and skeletal malocclusion in patients with AI has been observed in different populations. Open bite is the most commonly reported malocclusion^27^. In this study, the investigated AI patients showed various degrees of crowding, cross-bite, and tooth impaction, but no signs of obvious anterior open bite. It is notable that the nonsense mutation (c.973 C > T, p.R325X) identified in family 4 has been reported previously to cause hypocalcified AI in one Korean and two Chinese families, and is presumably an AI mutation hotspot in Asian population^6, 8, 28^. According to previous reports, several affected individuals in the Korean pedigree exhibited an anterior open bite; however, those in the Chinese pedigrees showed no signs of severe malocclusion. Although having the same AI-causing mutation, the proband in family 4 exhibited severe skeletal cross-bite and anterior crowding, which was quite different from the phenotypes of the reported cases.

To date, *FAM83H* has been discovered as the contributor to the aetiology of all reported hypocalcified AI cases. It is expressed in many tissues, while its mutations only cause phenotypes for dental enamel. The occurrence of truncating-only mutations in the last exon of *FAM83H* (except for one missense mutation) highly suggests a dominant negative effect or a gain of function effect as the underlying mechanism that triggers AI^7, 29^. Unlike the enamel matrix proteins and proteases important for enamel formation, FAM83H is a non-secreted protein because of the absence of a signal peptide^26^. Previous studies showed that wild-type FAM83H is localized in the cytoplasm, whereas the truncated FAM83H is expressed mainly in the nucleus^7^. It was shown that *Fam83h* overexpression did not cause an overt abnormality in enamel^30^. Moreover, inactivation of *Fam83h* expression in mouse models showed no apparent enamel defects^25^. These results further supported the pathological mechanism for hypocalcified AI in humans as a gain-of-function rather than a dominant negative effect^25^.

Recently, it has been revealed that FAM83H interacts with casein kinase I (CK-1) via an N-terminal motif, and has many phosphorylation sites in its C-terminus, which might be phosphorylated by CK-1^25^. *In vivo*, FAM83H and CK-1ε are co-localized on keratin filaments in mouse dental enamel cells. *In vitro*, FAM83H also showed preferential localization to keratin filaments around the nucleus, which often extended to cell-cell junctions in human ameloblastoma cells^24^. Furthermore, two AI-causing mutants of FAM83H (p.S287X, p.Y297X) were found to disturb the keratin cytoskeleton and disrupt desmosomes. The importance of keratins and desmosomal proteins during amelogenesis, taken together with our results, suggested that FAM83H might function as a scaffold protein to guide CK-1 to its physiological sites, where it is involved in the formation of enamel by regulating the organization of the keratin cytoskeleton^24^. In this study, similar results were found whereby the three novel FAM83H truncations exhibited a prominent nuclear localization in rat dental epithelial SF2 cells; however, wild-type FAM83H was distributed mainly around the nucleus and in the cytoplasm, with expression patterns similar to filamentous shapes. The alteration in the subcellular localization of FAM83H probably increased its concentration in the nucleus, while reducing the amount of FAM83H in the cytoplasm to a level below what is required for its normal function. The nuclear localization might prevent the proper organization of the keratin cytoskeleton and the formation of desmosomes via failing to recruit CK-1 to keratin filaments, thus leading to disturbed enamel mineralization. Otherwise, the ectopic location of mutant FAM83H in the nucleus might cause it to interact with nuclear proteins and interfere with their functions^7^.

The involvement of *FAM83H* in colorectal cancer was reported recently^31^. *FAM83H*-knockout mice model showed a slightly scurfy skin^25^. However, neither colorectal cancer nor skin problems have been reported in AI patients with *FAM83H* mutations, including the affected individuals in our study. The predicted structures of the N- and C-termini of FAM83H are quite different, indicating that the two parts may play different roles in different tissues^8^. Further studies are needed to understand the functions of FAM83H during enamel calcification and its bioactivities in other tissues.

## Materials and Methods

### Subjects and DNA extraction

The study protocol was reviewed and approved by the Ethics Committee of Peking University School and Hospital of Stomatology (PKUSSIRB-201311083). Informed consent was obtained from all participants, including the guardians on behalf of the minors enrolled, and all methods were in accordance with the Declaration of Helsinki. Four unrelated probands, without any other health problems, and their available family members, were recruited. Of these families, one showed dominant transmission, and three had only a single affected individual. There were no co-segregating systemic diseases reported in these families. According to previous studies, FAM83H is involved in colorectal cancer^31^. In addition, mild skin problems were also found in Fam83h null mice^25^. However, no history of such diseases was reported by the affected individuals, either. Each of the probands underwent comprehensive dental and radiological examinations. A 4 mL peripheral blood sample was obtained from each participant. Genomic DNA was extracted from the peripheral blood using the TIANamp Blood DNA mini kit (Tiangen, Beijing, China), according to the manufacturer’s instructions.

### Mutation analysis

The entire coding region and adjacent intron boundaries of the *FAM83H* gene were amplified by polymerase chain reaction (PCR) using Takara Ex-Taq (Takara Bio, Kyoto, Japan). Primers were designed using Primer 3 on the Web (http://bioinfo.ut.ee/primer3-0.4.0/). The products were purified and sequenced using an ABI 377 Automatic Sequencer (Applied Biosystems, Foster City, CA, USA). We analysed the insertion/deletion mutations with the help of Mutation Surveyor^®^ (SoftGenetics, State College, PA, USA).

### Cloning and mutagenesis of expression vectors

To transiently express green fluorescent protein fusions, the *FAM83H* coding sequencing was amplified from pCR2.1-FAM83H plasmid (kindly provided by Prof. Jan C-C. Hu, Department of Biologic and Materials Sciences, University of Michigan School of Dentistry) by PCR using forward (5′-GGAGATCTATGGCCCGTCGCTCTCAGAGCT-3′) and reverse (5′-GGAATTCACTTCTTGCTTTTGAACGTG-3′) primers containing *Bgl*II and *Eco*RI sites (underlined). The amplified product was subcloned into *Bgl*II and *Eco*RI sites of the pEGFP-C1 vector. The construct was completely sequenced to exclude random mutagenesis and was used as template for all other subcloning strategies. The mutants carrying V311Rfs*13 (forward, 5′-CGGGCCTCTCGTGGGC**C**GTCCCTGGGGTCGGGG-3′, reverse, 5′-CCCCGACCCCAGGGAC**G**GCCCACGAGAGGCCCG-3′), S377X (forward, 5′-GGGCTGCGGCCGCTCT**AA**CGGCGCCTGGAGGCCG-3′, reverse, 5′-CGGCCTCCAGGCGCCG**TT**AGAGCGGCCGCAGCCC-3′) and E383X (forward, 5′-GCGGCGCCTGGAGGCC**T**AGGCCGGGCCGGCTGG-3′, reverse, 5′-CCAGCCGGCCCGGCCT**A**GGCCTCCAGGCGCCGC-3′) mutations were constructed using the PrimeMut XL Site-directed Mutagenesis Kit (ExCell Biology, Shanghai, China), following the manufacturer’s instructions. Correct clones were confirmed by direct sequencing.

### Intracellular localization

Rat dental epithelial SF2 cells (kindly provided by Prof. Fukumoto S, Department of Oral Health and Development Sciences, Tohoku University Graduate School of Dentistry, Japan) were cultured in Dulbecco’s Modified Eagle Medium/F12 medium (DMEM-F12) supplemented with 10% foetal bovine serum (FBS) and 1% penicillin and streptomycin at 37 °C under 5% CO_2_. For transient transfection, SF2 cells were trypsinised, counted, and seeded on a coverglass in 6-well plates at a density of 8 × 10^4^ cells per well. After overnight incubation, 1.5 μg of plasmid DNA was transfected into the cells using Lipofectamine LTX (Invitrogen, Carlsbad, CA, USA), following the manufacturer’s instructions. Twenty-four hours after transfection, the cells were fixed with 4% paraformaldehyde, and nuclear DNA was stained in Hoechst 33342 (C1026, Beyotime, China) for 5 minutes. The samples were washed with phosphate buffered saline (PBS) three times for 5 minutes each. Confocal laser scanning was performed using a ZEISS-LSM 5 EXCITER fluorescence microscope. The experiment was performed in triplicate.

## Electronic supplementary material

Supplementary information
