# Supplementary material for: Novel FAM83H mutations in patients with amelogenesis imperfecta
Source: Sci Rep. 2017 Jul 20;7:6075. doi: 10.1038/s41598-017-05208-0 (PMC5519741; doi:10.1038/s41598-017-05208-0)
Supplement: Supplementary file 1 — Supplementary information [file 41598_2017_5208_MOESM1_ESM.pdf]

Novel *FAM83H* mutations in patients with amelogenesis imperfecta

Wang Xin, Wang Wenjun, Qin Man, Zhao Yuming

Supplementary information

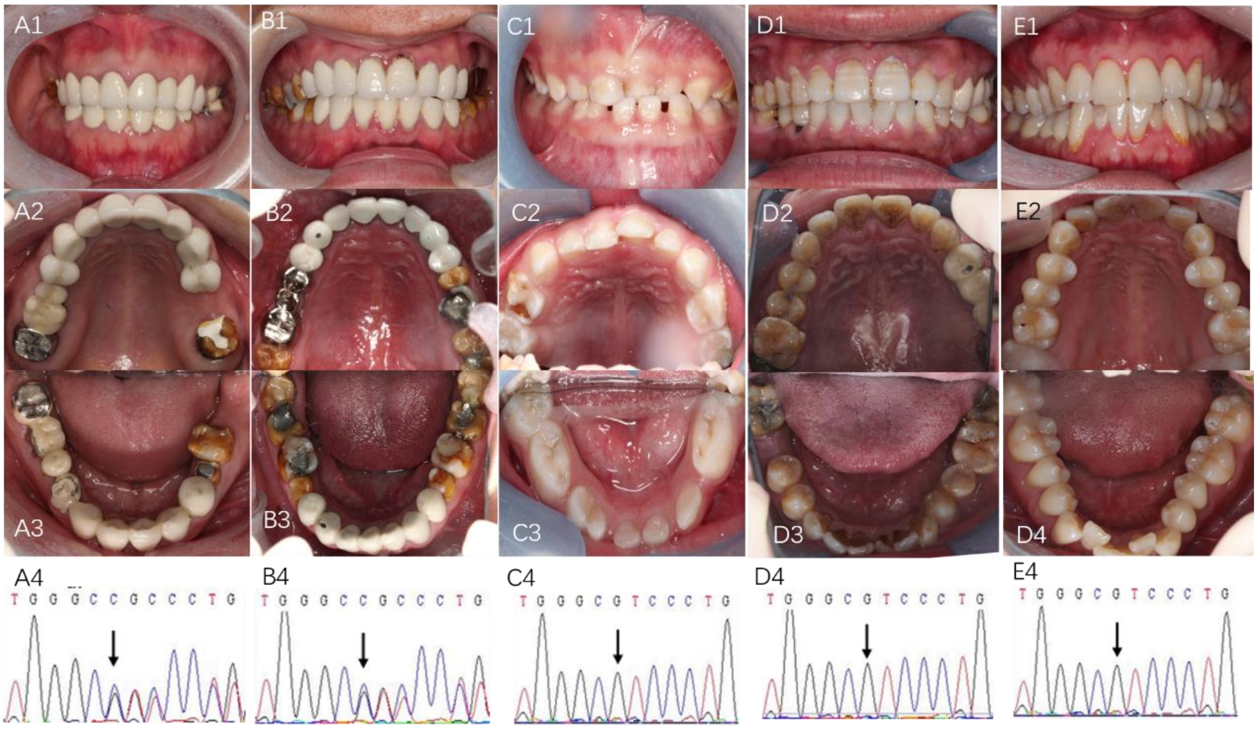

Figure S1. Family 1. Oral photographs and *FAM83H* exon 5 sequencing chromatograms of affected and unaffected family members. (A1-A4) the proband's mother (II:1) (B1-B4) the proband's affected uncle (II:6) (C1-C4) the proband's unaffected brother (III:2) (D1-D4) the proband's father (II:2) (E1-E4) the proband's unaffected uncle (II:7)

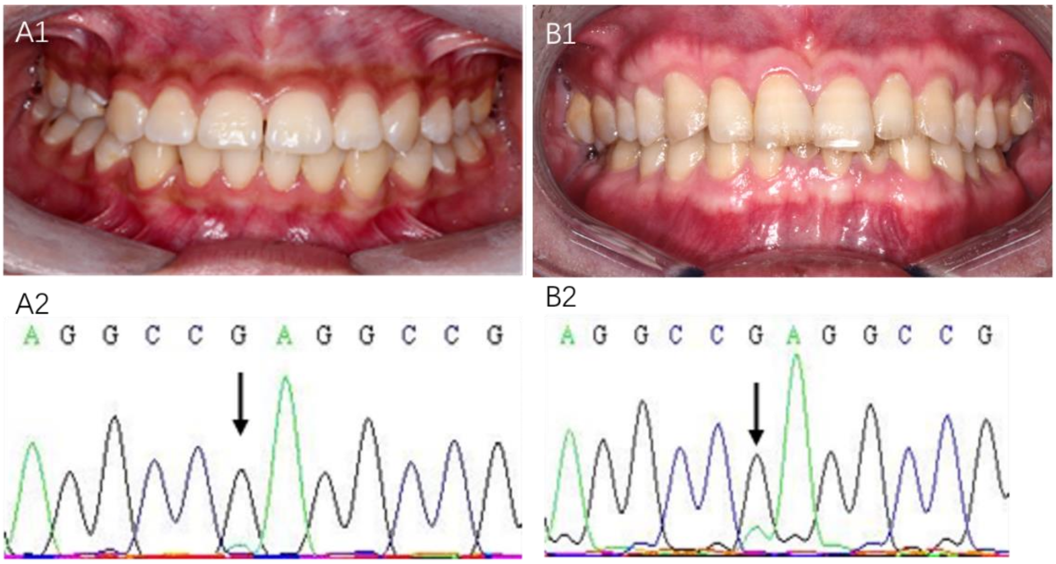

Figure S2. Family 2. Oral photographs and *FAM83H* exon 5 sequencing chromatograms of the proband's parents. (A1, A2) the proband's mother (II:1) (B1, B2) the proband's father (II:2)

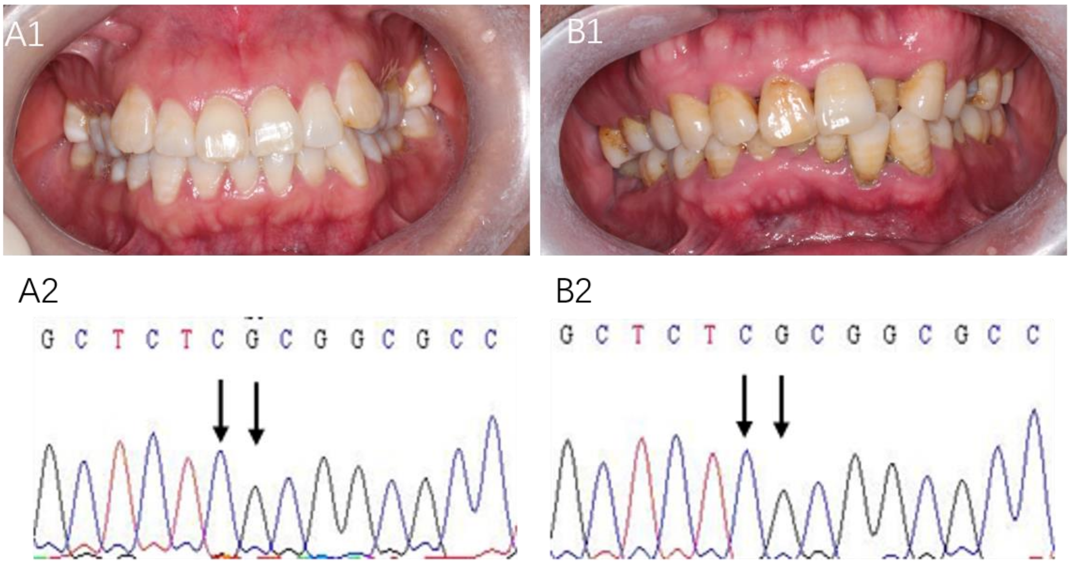

Figure S3. Family 3. Oral photographs and *FAM83H* exon 5 sequencing chromatograms of the proband's parents. (A1, A2) the proband's mother (I:1) (B1, B2) the proband's father (I:2)

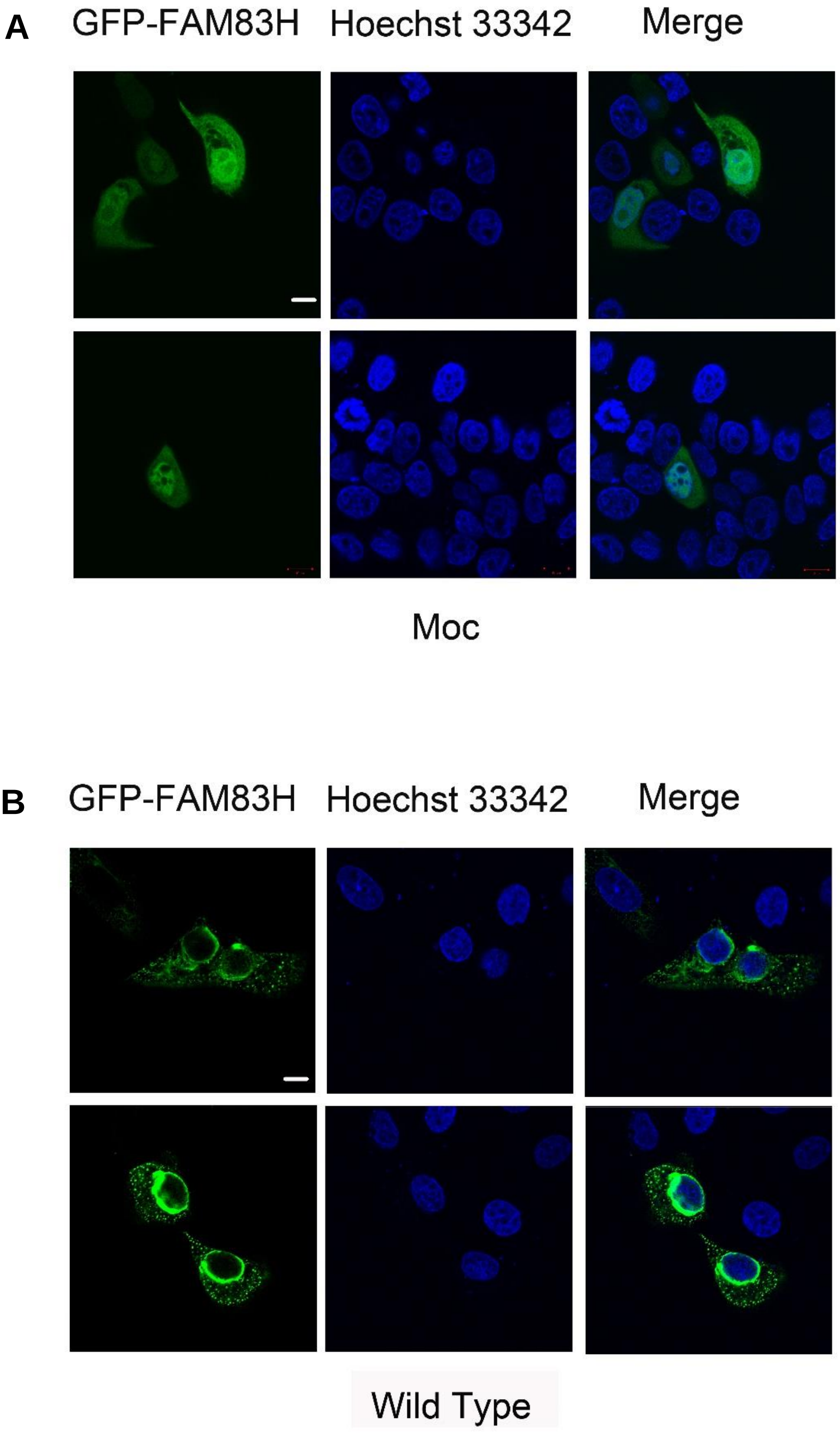
A
GFP-FAM83H
Hoechst 33342
Merge
Moc
B
GFP-FAM83H
Hoechst 33342
Merge
Wild Type

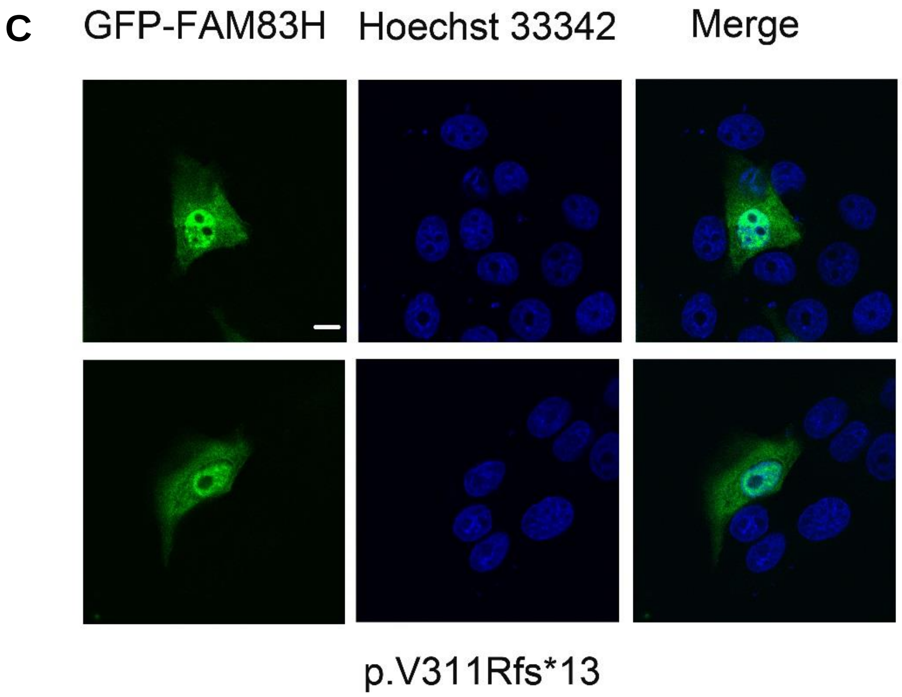
C
GFP-FAM83H
Hoechst 33342
Merge
p.V311Rfs*13

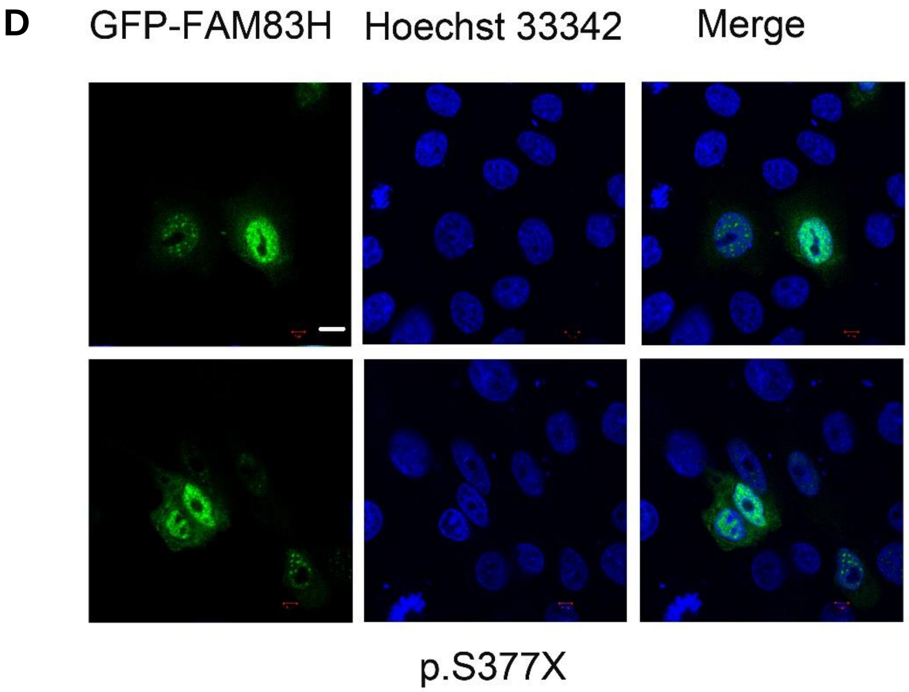
D
GFP-FAM83H
Hoechst 33342
Merge
p.S377X

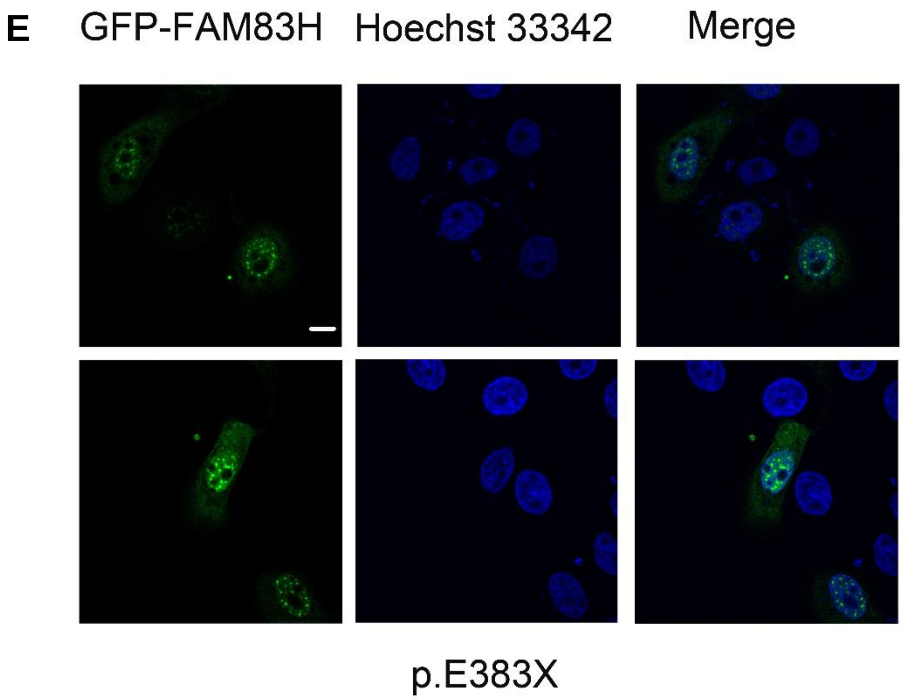

Figure S4. (A-E) Subcellular localization of wild-type and mutant FAM83H-GFP in SF2 cells. Figures picked from replicated experiments.
